# miR-455/GREM1 axis promotes colorectal cancer progression and liver metastasis by affecting PI3K/AKT pathway and inducing M2 macrophage polarization

**DOI:** 10.1186/s12935-024-03422-1

**Published:** 2024-07-05

**Authors:** Shipeng Dai, Fan Xu, Xiaozhang Xu, Tian Huang, Yiming Wang, Hongyu Wang, Yucheng Xie, Lei Yue, Wenhu Zhao, Yongxiang Xia, Jian Gu, Xiaofeng Qian

**Affiliations:** 1 Hepatobiliary Center, The First Affiliated Hospital of Nanjing Medical University, Key Laboratory of Liver Transplantation, Chinese Academy of Medical Sciences, NHC Key Laboratory of Living Donor Liver Transplantation (Nanjing Medical University), Nanjing, Jiangsu Province China; 2 Hangzhou Institute of Medicine (HIM), Zhejiang Cancer Hospital, Chinese Academy of Sciences, Hangzhou, Zhejiang China

**Keywords:** Colorectal cancer, GREM1, miR-455, PI3K, AKT, Liver metastasis, Macrophage polarization

## Abstract

**Background:**

Colorectal cancer is among the most common malignant tumors affecting the gastrointestinal tract. Liver metastases, a complication present in approximately 50% of colorectal cancer patients, are a considerable concern. Recently, studies have revealed the crucial role of miR-455 in tumor pathogenesis. However, the effect of miR-455 on the progression of liver metastases in colorectal cancer remains controversial. As an antagonist of bone morphogenetic protein(BMP), Gremlin 1 (GREM1) may impact organogenesis, body patterning, and tissue differentiation. Nevertheless, the role of miR-455 in regulating GREM1 in colorectal cancer liver metastases and how miR-455/GREM1 axis influences tumour immune microenvironment is unclear.

**Methods:**

Bioinformatics analysis shows that miR-455/GREM1 axis plays crucial role in liver metastasis of intestinal cancer and predicts its possible mechanism. To investigate the impact of miR-455/GREM1 axis on the proliferation, invasion, and migration of colorectal cancer cells, colony formation assay, wound healing and transwell assay were examined in vitro. The Dual-Luciferase reporter gene assay and RNA pull-down assay confirmed a possible regulatory effect between miR-455 and GREM1. In vivo, colorectal cancer liver metastasis(CRLM) model mice was established to inquiry the effect of miR-455/GREM1 axis on tumor growth and macrophage polarization. The marker of macrophage polarization was tested using immunofluorescence(IF) and quantitative real-time polymerase chain reaction(qRT-PCR). By enzyme-linked immunosorbent assay (ELISA), cytokines were detected in culture medium supernatants.

**Results:**

We found that miR-455 and BMP6 expression was increased and GREM1 expression was decreased in liver metastase compared with primary tumor. miR-455/GREM1 axis promotes colorectal cancer cells proliferation, migration, invasion via affected PI3K/AKT pathway. Moreover, downregulating GREM1 augmented BMP6 expression in MC38 cell lines, inducing M2 polarization of macrophages, and promoting liver metastasis growth in CRLM model mice.

**Conclusion:**

These data suggest that miR-455/GREM1 axis promotes colorectal cancer progression and liver metastasis by affecting PI3K/AKT pathway and inducing M2 macrophage polarization. These results offer valuable insights and direction for future research and treatment of CRLM.

**Supplementary Information:**

The online version contains supplementary material available at 10.1186/s12935-024-03422-1.

## Introduction

As one of the most common cancers of the gastrointestinal system and the third most common malignant tumor, colorectal cancer severely affects the lives and health of people [1–3]. The liver is the main place where colorectal cancer metastasizes. Around half of colorectal cancer patients develop liver metastases [4, 5]. Patients diagnosed with liver metastases typically face a bleak outlook, with median survival estimates ranging from 6 to 9 months without treatment and 13 to 18 months with optimal chemotherapy [6]. The preferred treatment for patients with resectable colorectal cancer liver metastases (CRLM) is primary tumor surgery combined with liver metastasis resection. It has been shown in previous research that about half of patients who undergo CRLM resection survive five years after the surgery, and about a quarter of patients survive ten years after the surgery [7, 8]. In view of this, it is vital to understand the mechanism how colorectal cancer metastasizes to the liver.

The microRNA (or miRNA for short) is a non-coding RNA molecule that has 22–24 nucleotides [9]. MiRNAs play dual roles as oncogenes and tumor suppressor genes by affecting the proliferation, migration, apoptosis, differentiation, metabolism, and metabolism of tumor cells. MiR-455 has been shown to promote cancer in breast cancer [10] and oral cancer [11], but suppress cancer in liver cancer [12] and medullary thyroid cancer [13]. In colorectal cancer, the role of miR-455 remains controversial, with some studies suggesting a cancer-promoting effect and others suggesting the opposite [14–16]. There is no report on the effects of miR-455 in the development of liver metastases in colorectal cancer.

Known as an antagonist of BMP, the gene Gremlin 1 (GREM1) regulates organogenesis, body patterning, and tissue differentiation [17, 18]. Tumor development is also influenced by GREM1. Breast cancer patients with high expression of GREM1 have a poor prognosis [19]. When GREM1 is overexpressed, glioma cells can multiply and metastasize, thus promoting the development of the disease [20]. Compared with the control group, the probability of liver metastasis in pancreatic cancer model KO-GREM1 mice was significantly increased [21]. In osteosarcoma cells, GREM1 is down-regulated. When osteosarcoma cells are overexpressed with GREM1, they are inhibited from proliferating, migrating, invading, and angiogenically growing [22]. Some studies have found that the high expression of GREM1 in intestinal epithelial cells can promote the occurrence of precancerous lesions of colorectal cancer [23]. However, another clinical study suggests that high expression of GREM1 is significantly associated with recurrence-free survival and overall survival, and is closely associated with low lymphatic and vascular infiltration and better prognosis [24]. The role of GREM1 in carcinogenesis and development of colorectal cancer is still vague, and the mechanism of GREM1 in liver metastasis of colorectal cancer has not been reported. Our study aims to explore the regulatory mechanism of miR-455/GREM1 axis on CRLM.

## Materials and methods

### Bioinformatics analysis

Three mRNA datasets (GSE41258, GSE49355, GSE81558) and one miRNA dataset (GSE81582) were obtained from GEO database. Differential expression of mRNAs and miRNAs were selected by GEO2R, an R-based online data processing tool on GEO.The inclusion criteria for differential expression of mRNAs and miRNAs were *p* < 0.05 and |log fold change (FC)| > 1. The Volcano plots were also drawn by GEO2R. The pheatmap R package was used to paint the heatmaps of 3 mRNA datasets by R language software 4.2.1. Then, a Venn plot (bioinformatics.psb.ugent.be/ webtools/Venn) was drawn using DEGs of the three datasets. FunRich software 3.1.3 has been used to predict mRNAs that may be regulated by differential miRNAs. PROGgeneV2 (http://genomics.jefferson.edu/proggene/) is an online software for analyzing the prognosis of malignant tumors. The high or low expression level groups were divided by using the median as the boundary according to the expression of crucial genes. The survival analysis was performed with a Kaplan-Meier curve, using PROGgeneV2. *P* < 0.05 has been set as the significance threshold. According to the DEGs obtained from the above three datasets, gene ontology (GO) and Kyoto Encyclopedia of Genes and Genomes (KEGG) annotations were performed using the Database for Annotation, Visualization and Integrated Discovery (DAVID) 6.8 (https://david.ncifcrf.gov/). The three mRNAs datasets were used for further Gene Set Enrichment Analysis (GSEA) by the gseaplot2 R package. The CIBERSORT R package was used for subsequent tumor immunoinfiltration analysis.We analyzed the relationship between target genes 8,217,175,982,171,759.

### Clinical specimens

Primary colorectal cancer tumour tissues, liver metastases and matched adjacent normal tissues of CRC were collected from 5 patients who underwent surgery in the First Affiliated Hospital of Nanjing Medical University. The selected patients had no other malignant tumors. Informed consent and approval were obtained from all the patients and the Ethics Committee of the first affiliated hospital of Nanjing Medical University(Approval NO: 2022-SRFA-310).

### Cell culture, transfection, and establishment of stable cell line

The human colon cancer cell lines (SW480, HCT116, SW620, LOVO, HT-29) and mouse colon adenocarcinoma cell line MC38 were purchased from the Shanghai Zhong Qiao Xin Zhou Biotechnology Co. Ltd (Shanghai, China). SW480, HCT 116 and MC38 cells were grown in DMEM medium (Keygen Biotech, China) supplemented with 10% fetal bovine serum (FBS) (Procell, China). Cells were maintained at 37℃ in a 5% CO2 incubator (Thermos, USA).

To perform overexpression experiments, miR-455 mimics or pc-GREM1 (Genechem, China) were transfected into SW480 cells and HCT116 cells using Lipofectamine 2000 reagent (11,668,019, Thermos, USA) according to the manufacturer’s instructions. Downregulation of GREM1 in MC38 cells was achieved via shRNA(Genechem, China), We cultured 1 × 10^5^ cells per well in a 6-well plate and 2 ml medium in an incubator for 24 h. The medium was then changed to 1 ml, and the appropriate amount of virus and 40 µl of polybrene was added to it (Sigma-Aldrich, USA). After 12–16 h of culture, the cells were cultured in normal medium and screened with purinomycin. RNA and proteins from cells were extracted after 48 h to verify the effect of transfection.

### Bone marrow-derived macrophage (BMDM) extraction and culture

The 6–8 weeks-old male C57BL/6 mice were selected in this study. The mice were housed under the following conditions: constant humidity and temperature at 22 °C, 12 h/12 h alternating light to simulate day and night, individually ventilated rearing cages, and unlimited dietary water.

After the mice were sacrificed without neck, the femur and tibia were separated, and the attached tissue was removed. The bone marrow was flushed into a 15 ml centrifuge tube with DMEM medium containing 100ng/ml M-CSF and 10% fetal bovine serum (FBS) until maturation and then centrifuged for 5 min at 1250 rpm. After removing the supernatant, 3 ml medium and red blood cell lysate was added and mixed and then centrifuged again. Finally, the supernatant was removed and the cells were resuspended for culture.

### Co-cultured system

BMDMs isolated from mice were co-cultured with the supernatant of MC38 cells which were transfected with the sh-GREM1 lentivirus or negative control for 3 days to induce the polarization. And then the functional makers of M1 or M2 macrophages were assayed.

### CRLM model mice establishment

All the animal studies were performed following the Guidelines for the Care and Use of Laboratory Animals and were approved by the Ethics Committee of Nanjing Medical University(Approval NO: 2022-SRFA-317). C57BL/6 male mice (6–8 weeks old) were selected for the experiments. After anesthesia, a transverse incision was made in the left upper abdomen, and the spleen was separated and exposed after the abdominal cavity was opened. A 1 ml syringe was used to inject 100 µl MC38 cells with a concentration of about 1 × 10^7^/ml into the splenic capsule at the lower pole of the spleen. When the splenic capsule at the injection site was white and swollen, the needle was pulled out, and the bleeding was stopped by pressure for two minutes. Then the abdomen was closed layer by layer. The liver tissues were harvested after 3 weeks and tumor numbers were recorded. The sample size of each experimental group was five.

Administration of clodronate-filled liposomes (CLD) or IL-10 inhibitor AS101 was before the construction of CRLM model mice. From one day before modeling, 200 µl/mouse CLD was injected into the mice by intraperitoneal injection at 3-day intervals, while AS101 was injected 10ug/mouse intraperitoneally every day, until the end of modeling.

### qRT-PCR

Total RNA contents from liver metastasis, CRC tissues, or cells were detected by using a total RNA extraction kit (Yishan Biotech, China) according to the manufacturer’s instructions. The first strand of cDNA was synthesized using the GoScript Reverse Transcription System (Promega, USA). And the qRT-PCR analyses (10 µl) were performed according to the ratio of pure water: SYBR Green (Vazyme, China): pre-primer: post-primer: cDNA = 3.6:5.0:0.2:0.2:1.0. GAPDH was used as an internal control. The relative levels of RNA were calculated by the 2^−∆∆Ct^ method. All the primers were purchased from Sangon Biotech Co. Ltd (Shanghai, China) (Table S1).

### Western blot analysis

The treated cells were collected, and RIPA lysis buffer(Servicebio, China) was employed to extract the total protein. The protein concentration was determined by using Bicinchoninic Acid (BCA) method. The same amounts of proteins were separated in SDS-PAGE (Epizyme, China) and then transferred to PVDF membranes (Millipore, USA). The membranes were blocked with 5% skim milk powder solution for 1 h at room temperature and then incubated with primary antibodies at 4℃ overnight. After rinsing in TBST, the membranes were incubated with HRP-labeled secondary antibody (1:2000) at room temperature for 2 h. We uesd the following primary antibodies: GREM1( (1:1000, Abcam), BMP6 (1:1000, Abcam), phosphorylated PI3K (p-PI3K, 1:2000, Abcam, USA), PI3K (1:1000, Abcam), phosphorylated AKT (p-AKT, 1:1000, Abcam), AKT (1:500, Abcam), and GAPDH (1:5000, Proteintech) were used. All antibodies were diluted in the appropriate proportions using an antibody diluent (New Cell & Molecular Biotech Co.Ltd).

### ELISA

GREM1, BMP6, IL-1β, TNF-a, IL-10, and TGF-β levels in supernatant were measured using commercially available ELISA kits (MultiSciences, China) according to the manufacturer’s instructions. Analysis was performed using an microplate reading element, measuring the absorbance (OD) of the sample at 450 nm.

### Immunofluorescence assay

Paraffin sections of liver metastasis from mice were dewaxed, antigen repaired and serum blocked. After incubated with primary antibodies at 4 °C overnight, sections were incubated with secondary antibodies one hour at room temperature. Sections were incubated with bovine serum albumin(Servicebio, China) for 10 min at room temperature in the dark after washed with PBS (Gibco, USA), followed by three times washes with TBST. Sections were reverse stained with 4’,6-diamidino-2-phenylindolem (Servicebio, China), sealed, and finally, the images were observed under a fluorescent microscope(Zeiss, Germany).

### Colony formation assay

Before incubated at 37℃ with 5% CO_2_ for 10 days until the colonies were visible, 1 × 10^3^ SW480 and HCT116 cells were seeded into per well of 6-well plates. After washed with PBS (Gibco, USA), The colonies fixed with 4% paraformaldehyde(Servicebio, China) for 30 min, then washed again with PBS, and finally stained with crystal violet (Beyotime Biotechnology, China) for half an hour. Each well was photographed after staining, and the Image J software was used to calculate the colony numbers.

### Wound healing assay

8 × 10^5^ SW480 and HCT116 cells were seeded into per well of 6-well plates. When confluence reached > 90%, a scratch was created on the cell monolayer by a 10ul sterile pipette. The cells were washed with PBS and cultured in a DMEM medium supplemented with 10% FBS. Open wound areas were photographed with a microscope at 0, 24, or 48 h. The Image J software was used to analyze the percentage reduction of the open wound area at different time points compared with the initial scratch (0 h).

### Transwell assay

Transwell assay was performed by transwell chambers (3422, Corning USA) which were placed into a 24-well plate. 2 × 10^4^ SW480 and HCT116 cells were seeded into the upper chamber and cultured in 200ul serum-free DMEM medium. The lower wells were added 800ul DMEM medium containing 10% FBS. After incubating at 37℃ for 24 h, PBS was used to wash the transwell chambers. 4% paraformaldehyde fixed the transwell chambers for 30 min. Before finally stained with crystal violet for half an hour, these transwell chambers washed again with PBS. Each transwell chamber was photographed with a microscope, and then the number of stained cells in the image was measured by Image J software.

### Dual-luciferase reporter assay

Wild-type(WT) and mutant(MUT) GREM1 plasmids were designed. The WT or MUT vectors were cotransfected into SW480 and HCT116 cells cells with miR-455 mimics using transfection reagents, respectively. Then, the relative luciferase activity was examined by Dual Luciferase Assay Kit (Promega, USA) according to the manufacturer’s protocol.

### Pull-down experiment

A biotin-labeled RNA probe specific to miR-455 was synthesized (RiboBio, China). SW480 cells were cross-linked with 1% formaldehyde and lysed in a lysis buffer, total RNA was isolated and then incubated with 100 nmol miR-455-specific probe, followed by binding to 50 µL Streptavidin Magnetic Beads lysates to capture the biotin-labeled RNA-protein complexes. Streptavidin Magnetic Beads were then washed three times, and complexes were isolated. RNAbinding-protein was assessed by qRT-PCR and Western blotting.

### Statistical analysis

GraphPad Prism 9.0 software was uesd to perform all statistical analyses. All data were expressed as mean ± standard error (SD) and analyzed by a Student’s t-test for the comparison between two groups, and one-way analysis of variance (ANOVA) for univariate comparison. The Pearson correlation analysis was used to measure the correlation between two different variables. *p* < 0.05 indicated a statistically significant difference (*p < 0.05, **p < 0.01, ***p < 0.001).

## Results

### Differential expression of mRNAs and miRNAs in liver metastasis(LM) and primary tumor(PT) of CRC

**Fig. 1:**
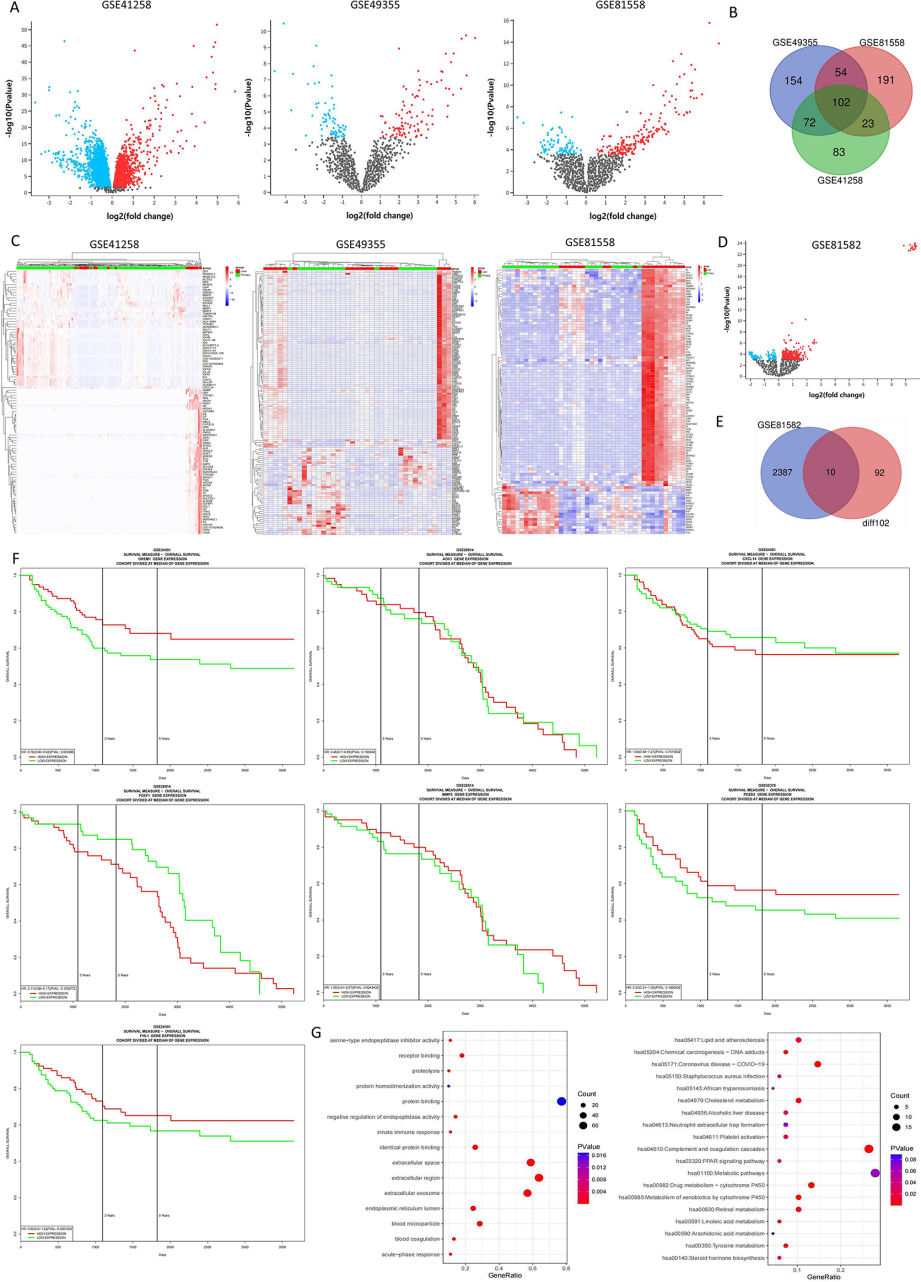
Differential expression of mRNAs and miRNAs between LM and primary tumors (PT) of CRC. (**A**) Volcano plots for DEGs from three mRNA datasets (GSE41258, GSE49355, GSE81558). (**B**) The Venn diagram for consistent total DEGs. (**C**) Heatmaps for DEGs of three mRNAs datasets. (**D**) Volcano plots for differential expression of miRNAs from GSE81582. (**E**) The Venn diagram of the predicted mRNAs and above differential mRNAs. (**F**) The KM survival analysis of seven key genes. (**G**) GO (Biological process, Cellular component, and Molecular function) and KEGG pathway enrichment analysis of consistent DEGs

With a view to study the difference of mRNAs and miRNAs expression between LM and PT of CRC, we selected three mRNA datasets (GSE41258, GSE49355, GSE81558) and one miRNA dataset (GSE81582) in GEO database. The Venn diagram shows a total of 102 consensus genes, of which 29 mRNAs expression increased and 73 mRNAs expression decreased (Fig. 1B, Fig S1). After analyzing the miRNA dataset, we obtained 60 differential miRNAs(Fig. 1D, Fig S2). FunRich software was used to predict the downstream mRNAs, and finally 2397 downstream mRNAs were gained. Ten groups of miRNA/mRNA axes which may have regulatory relationship were acquired by intersecting the predicted mRNAs with the above 102 differential mRNAs(Fig. 1E). Because miRNA often negatively regulates mRNA expression, we excluded three groups of non-negatively regulated axes, and finally received seven groups of miRNA/mRNA axes: miR-106b/MMP2, miR-339/FHL1, miR-92b/PDZD2, miR-133a/AOX1, miR-106b/CXCL14, miR-455/GREM1, miR-93/FOXF1. The differentially expressed mRNAs and miRNAs from each dataset are shown in Fig. 1A. The DEGs created by heatmap are exhibited in Fig. 1C. Subsequently, we investigated the correlation between the seven genes and CRC prognosis by using PROGgeneV2. The KM survival analysis indicated that low content of GREM1 was related to poor overall survival (OS) (*P* = 0.003886) (Fig. 1F). GO functional analysis revealed that these DEGs were obviously related to protein binding, extracellular region and extracellular space while KEGG enrichment of DEGs mainly involved Metablic pathways(Fig. 1G).

### Prediction of GREM1 downstream pathway and its effect on tumor immune microenvironment

**Fig. 2:**
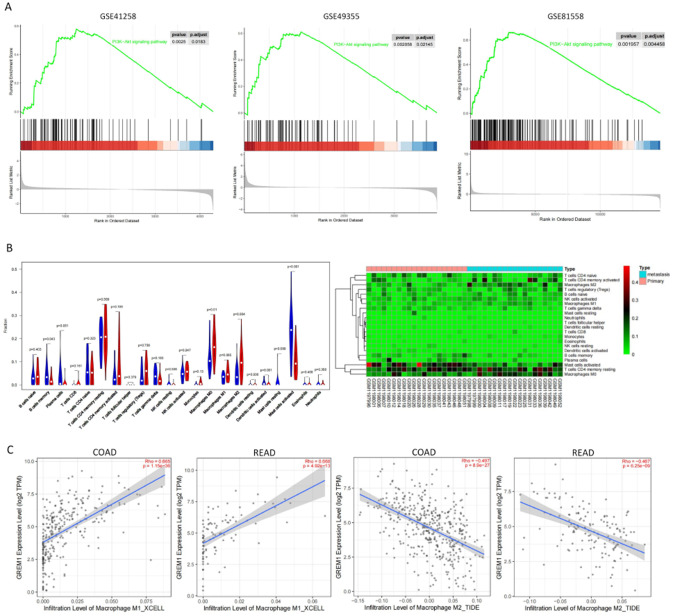
Prediction of GREM1 downstream pathway and its effect on tumor immune microenvironment. (**A**) Gene sets enrichment analyses of three mRNAs datasets. (**B**) immune infiltration analysis of GSE49355 dataset. (**C**) Differential Expression of GREM1 affect Macrophages Polarization according to TIME database

The gene sets enrichment analyses revealed that liver metastasis of colorectal cancer was significantly associated with the PI3K/AKT signaling pathway across three independent datasets (GSE41258, GSE49355, and GSE81558) (Fig. 2A). Using the GSE49355 dataset, we further investigated immune infiltration and observed that M2 polarization of macrophages was more pronounced in LM compared to primary tumors (PT) (Fig. 2B). The analysis of data from the TIME database revealed a positive correlation between GREM1 expression in colorectal cancer tissues and M1 polarization of macrophages, as well as a negative correlation with macrophage M2 polarization (Fig. 2C). Collectively, our findings suggest that low expression of GREM1 may contribute to the progression and metastasis of colorectal cancer via the PI3K/AKT pathway and by influencing macrophage polarization.

### Expression of miR-455, GREM1 and BMP6 in colorectal cancer, adjacent tissues and liver metastasis

**Fig. 3:**
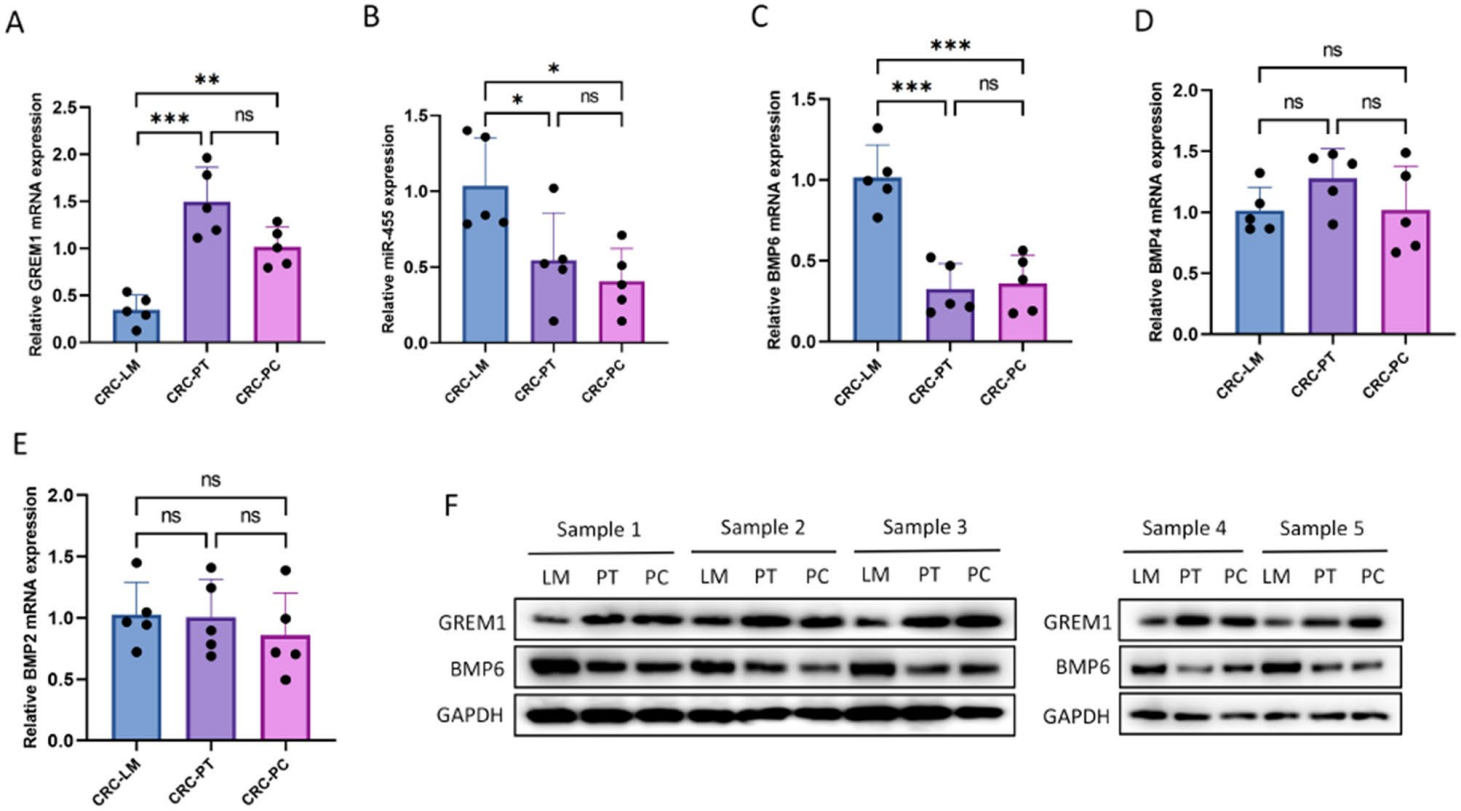
GREM1 expression is downregulated in LM of CRC.(**A**-**E**) The expression level of GREM1, miR-455, BMP6, BMP4, BMP2 in the liver metastases, primary tumors of CRC patients, and the adjacent normal tissues detected by qRT-PCR. (**F**) The expression level of GREM1, BMP6, in the liver metastases, primary tumors of CRC patients, and the adjacent normal tissues detected by WB

For investigate the expression of miR-455、GREM1 and BMP6 in colorectal cancer, we collected LM tissues, PT tissues and PC tissues of colorectal cancer of 5 patients for qRT-PCR and western blotting detection from the First Affiliated Hospital of Nanjing Medical University. The qRT-PCR analysis results showed that the expression of miR-455 (Fig. 3B) and BMP6 (Fig. 3C) in LM was higher than that in PT, while the expression of GREM1 (Fig. 3A) was lower. There was no statistically significant difference in the expression of BMP4 (Fig. 3D) and BMP2(Fig. 3E) between LM and PT. The difference of GREM1 (Fig. 3A) and miR-455 (Fig. 3B) was not found between PT tissue and PC tissue. Compared with PT, the expression of GREM1 in LM decreased and the expression of BMP6 increased by WB analysis (Fig. 3F).

### miR-455 directly targeted GREM1 to promote the proliferation and migration of colorectal cancer cells by affecting PI3K/AKT pathway

**Fig. 4:**
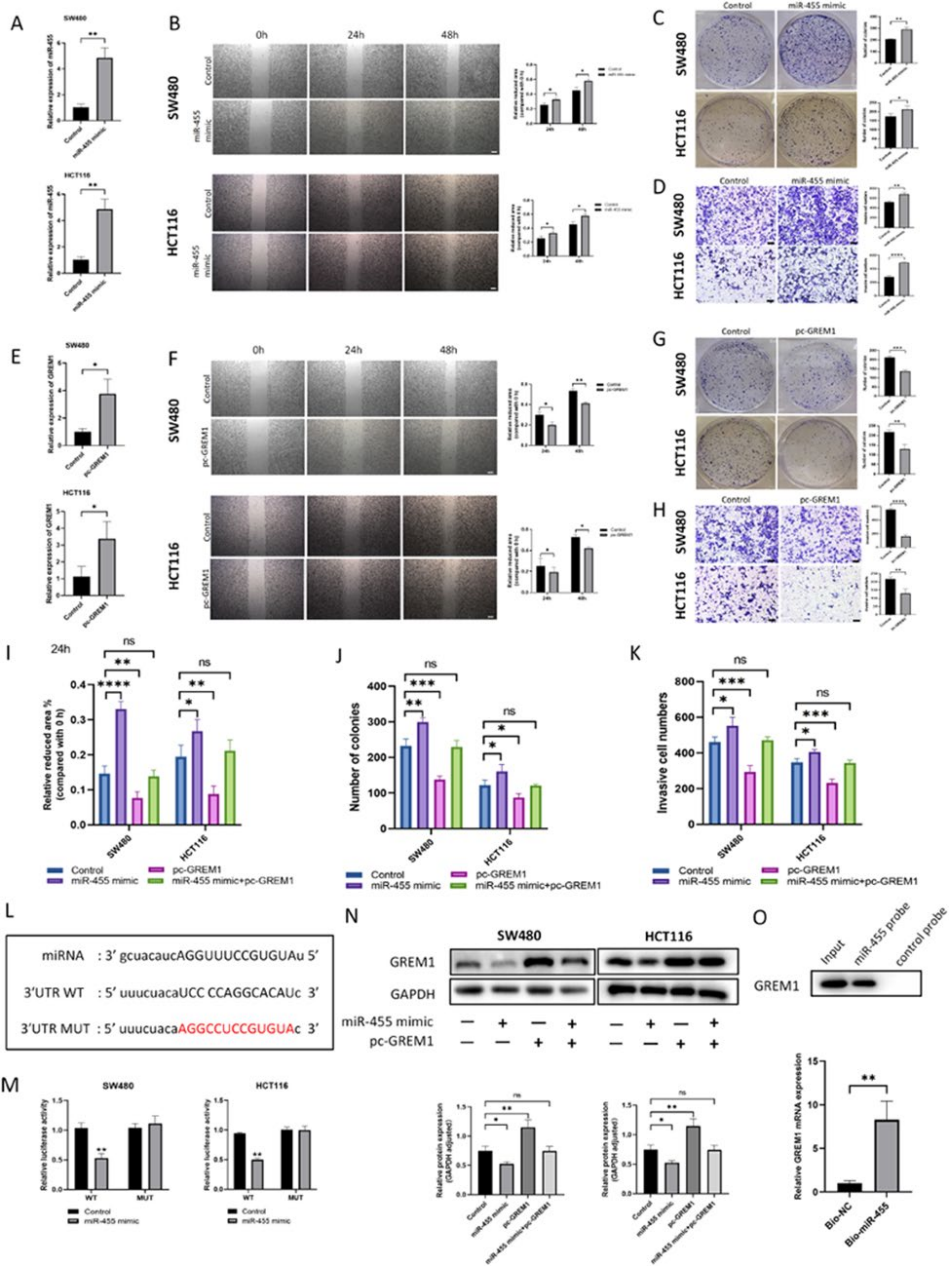
The effect of miR-455 on the human colorectal cancer cells by targeting GREM1. (**A**) qRT-PCR analysis of miR-455 or GREM1 expression in human colorectal cancer cells overexpressing miR-455 or control. (**B**) Analysis of human colorectal cancer cells overexpressing miR-455 or control migration by wound-healing assays at 0, 24, and 48 h. (**C**) The colony formation assay was performed to evaluate the proliferation of human colorectal cancer cells overexpressing miR-455 or control. (**D**) The migration of miR-455 overexpressing or control human colorectal cancer cells were assessed using Transwell migration assays. (**E**) qRT-PCR analysis of GREM1 expression in human colorectal cancer cells overexpressing GREM1 or control. (**F**) Analysis of human colorectal cancer cells overexpressing GREM1 or control migration by wound-healing assays at 0, 24, and 48 h. (**G**) The colony formation assay was performed to evaluate the proliferation of human colorectal cancer cells overexpressing GREM1 or control. (**H**) The migration of GREM1 overexpressing or control human colorectal cancer cells were assessed using Transwell migration assays. (**I**) Analysis of human colorectal cancer cells of control group, miR-455 mimic group, pc-GREM1 group and miR-455 mimic + pc-GREM1 group migration by wound-healing assays at 0, 24, and 48 h. (**J**) The colony formation assay was performed to evaluate the proliferation of human colorectal cancer cells of control group, miR-455 mimic group, pc-GREM1 group and miR-455 mimic + pc-GREM1 group. (**K**) The migration of control group, miR-455 mimic group, pc-GREM1 group and miR-455 mimic + pc-GREM1 group was assessed using Transwell migration assays. (**L**) TargetScan predicted that GREM1 was a potential target of miR-455. (**M**) miR-455 mimic reduced luciferase ability of GREM1 wild-type mRNA 3 ‘-UTR, while it did not change GREM1 mutant 3’-UTR. (**N**) GREM1 protein expression level of control group, miR-455 mimic group, pc-GREM1 group and miR-455 mimic + pc-GREM1 group migration were detected by Western blot. (**O**) The expression level of GREM1 in RNA pull-down test was detected by Western blot assay and qRT-PCR analysis

TargetScan predicts miR-455 directly targets 3 ‘-UTR of GREM1 mRNA(Fig. 4L). To verify the binding of miR-455 to 3 ‘-UTR of GREM1 mRNA, we co-transfected miR-455 mimic with GREM1 wild-type 3’-UTR or GREM1 mutant 3’-UTR in human colorectal cancer cells, and then measured and calculated luciferase ability. We found that miR-455 mimic reduced luciferase ability of GREM1 wild-type mRNA 3 ‘-UTR (P < 0.05), while it did not change GREM1 mutant 3’-UTR (P > 0.05) (Fig. 4M). According to WB analysis, GREM1 expression was decreased in miR-455 mimic group, and up-regulated in pc-GREM1 group. Compared with miR-455 mimic group, GREM1 expression was significantly increased in miR-455 + pc-GREM1 group, indicating successful transfection, and miR-455 targeting down-regulated GREM1 expression (Fig. 4N). A pull-down assay revealed that GREM1 could be pulled down by the miR-455 probe (Fig. 4O).

**Fig. 5:**
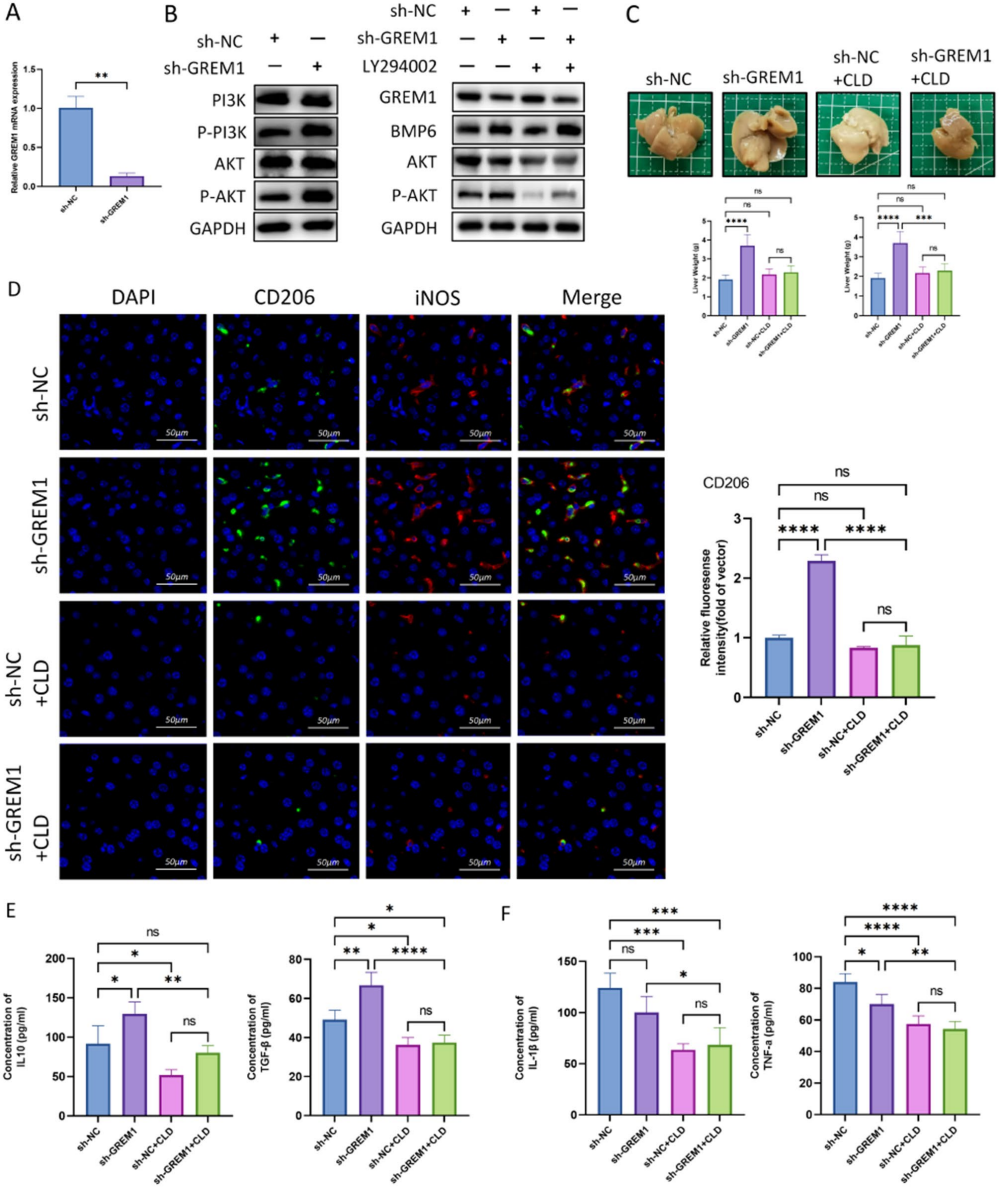
Deletion of GREM1 promoted liver metastasis of colorectal cancer and induced the M2 polarization of macrophages. (**A**) qRT-PCR analysis of GREM1 expression in MC38 cells of sh-NC and sh-GREM1 group. (**B**) Western blot assay was performed to detect the level of GREM1, BMP6, p-PI3K, PI3K, p-AKT, AKT in MC38 cells of different groups. (**C**) The representative images of liver metastases from the sh-NC or sh-GREM1 injected mice treated with CLD or not. Statistical results of the detectable tumor numbers of liver metastasis and liver weight. (**D**) Immunofluorescence staining for CD206 and iNOS from metastatic lesions. Statistical results of immunofluorescence staining for CD206. (**E**) The expression levels of IL-10,TGF-β in metastatic nodules of the liver were measured by ELISA. (**F**) The expression levels of IL-1β, TNF-a in metastatic nodules of the liver were measured by ELISA

In order to investigate the influence of miR-455/GREM1 axis on the progression and metastasis of colorectal cancer, we used miR-455 mimic and GREM1 overexpression plasmids to study in vitro (Fig. 4A). In clone formation experiments, we found that miR-455 mimic group showed the stronger cell proliferation rate and colony formation number compared with control group, while PC-GREM1 group showed the poorer cell proliferation ability (Fig. 4C and G). We used scratch test and Transwell test to evaluate cell proliferation, migration and invasion ability. As expected, miR-455 group showed the larger migration area at 24 h and 48 h compared with 0 h, while PC-GREM1 group showed the smaller migration area (Fig. 4B and F). Transwell experiment also showed the same trend (Fig. 4D and H). miR-455 mimic + PC-GREM1 group and control group had no noteworthy difference on the proliferation, invasion, and migration of human colorectal cancer cells(Fig. 4I-K). Therefore, we believe that miR-455 targets GREM1 to promote the proliferation and migration of colorectal cancer cells. The results of bioinformatics analysis suggest that PI3K-AKT signaling pathway plays an important role in CRLM (Fig. 2A), In the following experiments, we found that when GREM1 was knocked down in the MC38 cells, the content of p-PI3K and p-AKT was significantly increased, but the protein concentration of PI3K and AKT showed no obvious changes (Fig. 5B). Moreover, we used LY294002, a broad-spectrum PI3K inhibitor, to perform experimental intervention. The experimental results showed that LY294002 partially reversed the increased p-AKT expression elicited by knockdown of GREM1(Fig. 5B). These results illustrate that knocking down GREM1 can promote the development of colorectal cancer by activating phosphorylation of PI3K/AKT pathway.

### Deletion of GREM1 promoted liver metastasis of colorectal cancer and induced the M2 polarization of macrophages

To investigate the influence of GREM1 on macrophage polarization in tumor microenvironment, we transfected MC38 cells with lentivirus knocking down GREM1 or empty vector(MC38/sh-GREM1 or MC38/sh-NC). qRT-PCR and WB had been used to investigate the transfection efficiency (Figs. 3B and 5A). Subsequently, we used lentivirus-transfected MC38 cells to establish four groups of CRLM models in mices. Two groups were treated with CLD to eliminate macrophages. Four weeks later, liver specimens were collected and liver metastases were counted. These outcomes manifested that the number of metastatic tumors in LM/sh-GREM1 group was significantly higher than that in LM/sh-NC group and the liver weight in LM/sh-GREM1 group was larger than that in LM/sh-NC group, and the number of metastatic tumors and liver weight in LM/sh-NC + CLD group and LM/sh-GREM1 + CLD group was less than that in LM/sh-NC group and LM/sh-GREM1 group, respectively (Fig. 5C). This suggests that knocking down GREM1 can promote the occurrence of CRLM, which may related to its induction of macrophage polarization.

It is well known that the polarization of macrophages is closely associated with the occurrence and development of diseases. Current researchs suggest that M2 polarization of macrophages promotes tumor progression. Therefore, we used immunofluorescence(IF) to detect mice liver metastases and found that the expression of macrophage M2 polarization marker CD206 was increased in LM/sh-GREM1 group, and the expression of CD206 was significantly inhibited in CLD treated mice liver metastases (Fig. 5D). ELISA results showed that IL-10 and TGF-β secreted by M2 increased in LM/sh-GREM1 group, while TNF-α secreted by M1 decreased (Fig. 5E and F).

### Knocking down GREM1 promotes M2 polarization of macrophages via increasing BMP6 expression

**Fig. 6:**
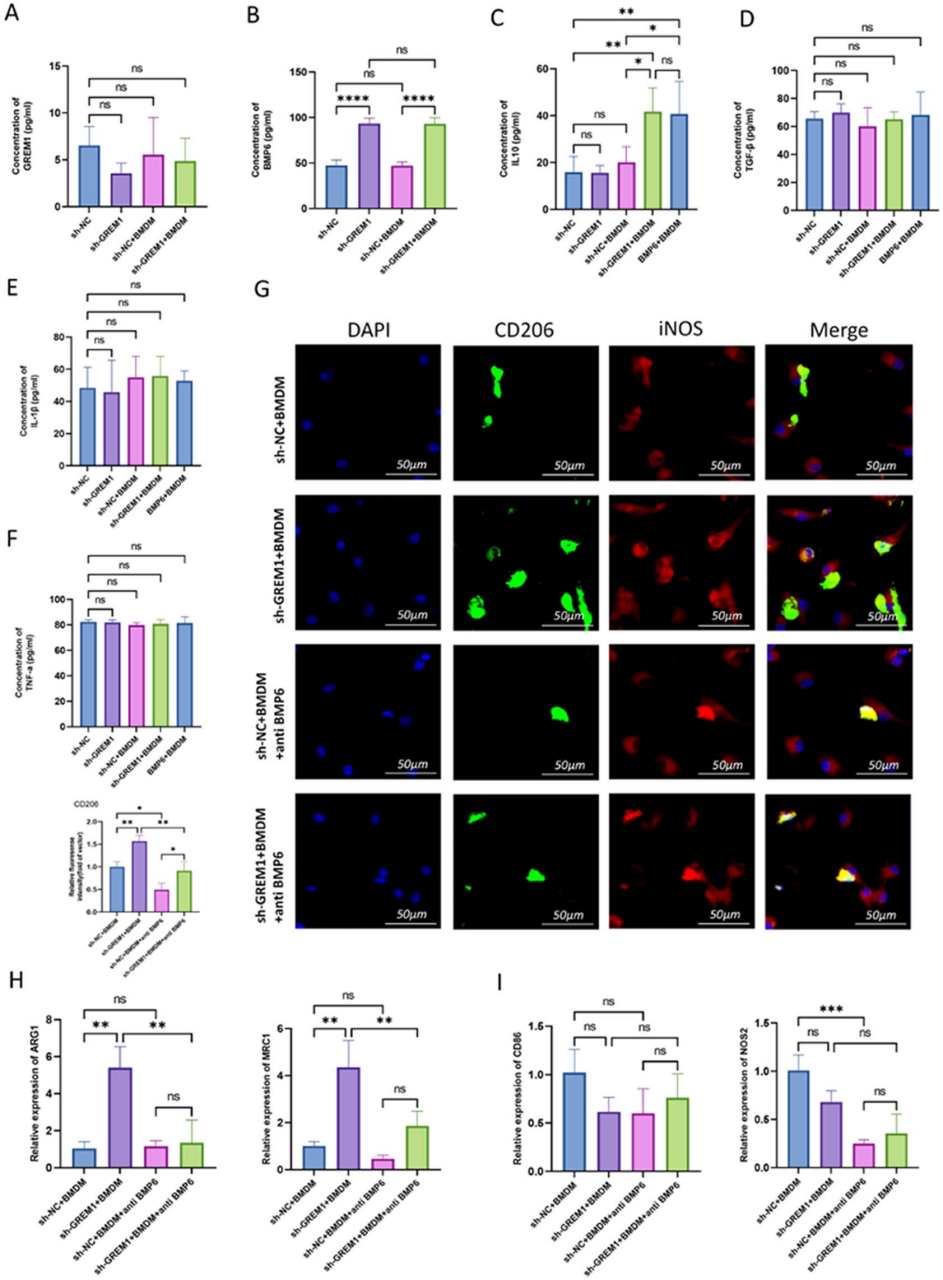
Knocking down GREM1 promotes M2 polarization of macrophages via increasing BMP6 expression. (**A**-**F**) The levels of GREM1, BMP6, IL-10, TGF-β, IL-1β, TNF-a in the supernatant of sh-NC, sh-GREM1, sh-NC + BMDM, sh-GREM1 + BMDM, BMP6 + BMDM group measured by ELISA. (**G**) Immunofluorescence staining for CD206 and iNOS from BMDMs co-cultured with MC38/sh-GREM1(sn), MC38/sh-NC(sn), MC38/sh-GREM1(sn) + anti-BMP6 neutralizing antibodies, MC38/sh-NC(sn) + anti-BMP6 neutralizing antibodies. (**H**) Representative histograms of the relative mRNA levels of ARG1, MRC1 for BMDMs treated with MC38/sh-GREM1(sn), MC38/sh-NC (sn), MC38/sh-GREM1(sn) + anti-BMP6 neutralizing antibodies, MC38/sh-NC(sn) + anti-BMP6 neutralizing antibodies. (**I**) Representative histograms of the relative mRNA levels of CD86, NOS2 for BMDMs treated with MC38/sh-GREM1(sn), MC38/sh-NC (sn), MC38/sh-GREM1(sn) + anti-BMP6 neutralizing antibodies, MC38/sh-NC (sn) + anti-BMP6 neutralizing antibodies

For the sake of exploring the mechanism that knocking down GREM1 promotes M2 polarization of macrophages, we detected the contents of GREM1, BMP6 and cytokines in the supernatant(sn) of MC38/sh-NC, MC38/sh-GREM1, MC38/sh-NC + BMDM and MC38/sh-GREM1 + BMDM dishes by ELISA. We found that BMP6 levels in MC38/sh-GREM1 (sn) were higher than those in MC38/sh-NC (sn)(Fig. 6B), but there was no statistical difference in the expression levels of GREM1, TNF-α, TGF-β and IL-1β between these groups(Fig. 6A and D-F). It can be seen that the inhibition of GREM1 expression has no obvious effect on the secretion of cytokines in MC38 cells(Fig. 6C-F). BMDM extracted from mice was co-cultured with MC38/sh-GREM1 (sn) and MC38/sh-NC (sn) for 3 days, and then the expression level of cytokines in the supernatants was detected again. The results showed that the expression of IL-10 in the supernatant of BMDM co-cultured with MC38/sh-GREM1 (sn) was significantly increased. 100 ng/mL of BMP6 also increased the expression of IL-10 in BMDM medium, and its effect was equivalent to that of knocking down GREM1(Fig. 6C).

In addition, we collected primary BMDM extracted from mice and co-cultured it with MC38/sh-GREM1 (sn), MC38/sh-NC (sn), MC38/sh-GREM1 (sn) + anti-BMP6 neutralizing antibodies, MC38/sh-NC (sn) + anti-BMP6 neutralizing antibodies. After 3 days, IF and qRT-PCR were used to detect the polarization of BMDM. IF shows that, compared with BMDM co-cultured with MC38/sh-NC (sn), BMDM co-cultured with MC38/sh-GREM1 (sn) had higher expression of CD206 but no significant difference was found in iNOS expression. CD206 expression of BMDM co-cultured with MC38/sh-NC (sn) + anti-BMP6 neutralizing antibodies was lower than the group co-cultured with MC38/sh-NC (sn). anti-BMP6 neutralizing antibodies also eliminated the promotion of knocking down GREM1 on CD206 expression in BMDM(Fig. 6G). qRT-PCR showed that BMDM co-cultured with MC38/sh-GREM1 (sn) had higher expression of M2 markers, including ARG1 and MRC1, than those co-cultured with MC38/sh-NC (sn)(Fig. 6H); While M1 markers NOS2 decreased(Fig. 6I). These data suggest that knocking down GREM1 can increase BMP6 secretion and thus promote the preferential polarization of macrophages to M2.

#### BMP6 promotes M2 polarization by inducing IL-10 release from macrophages

**Fig. 7:**
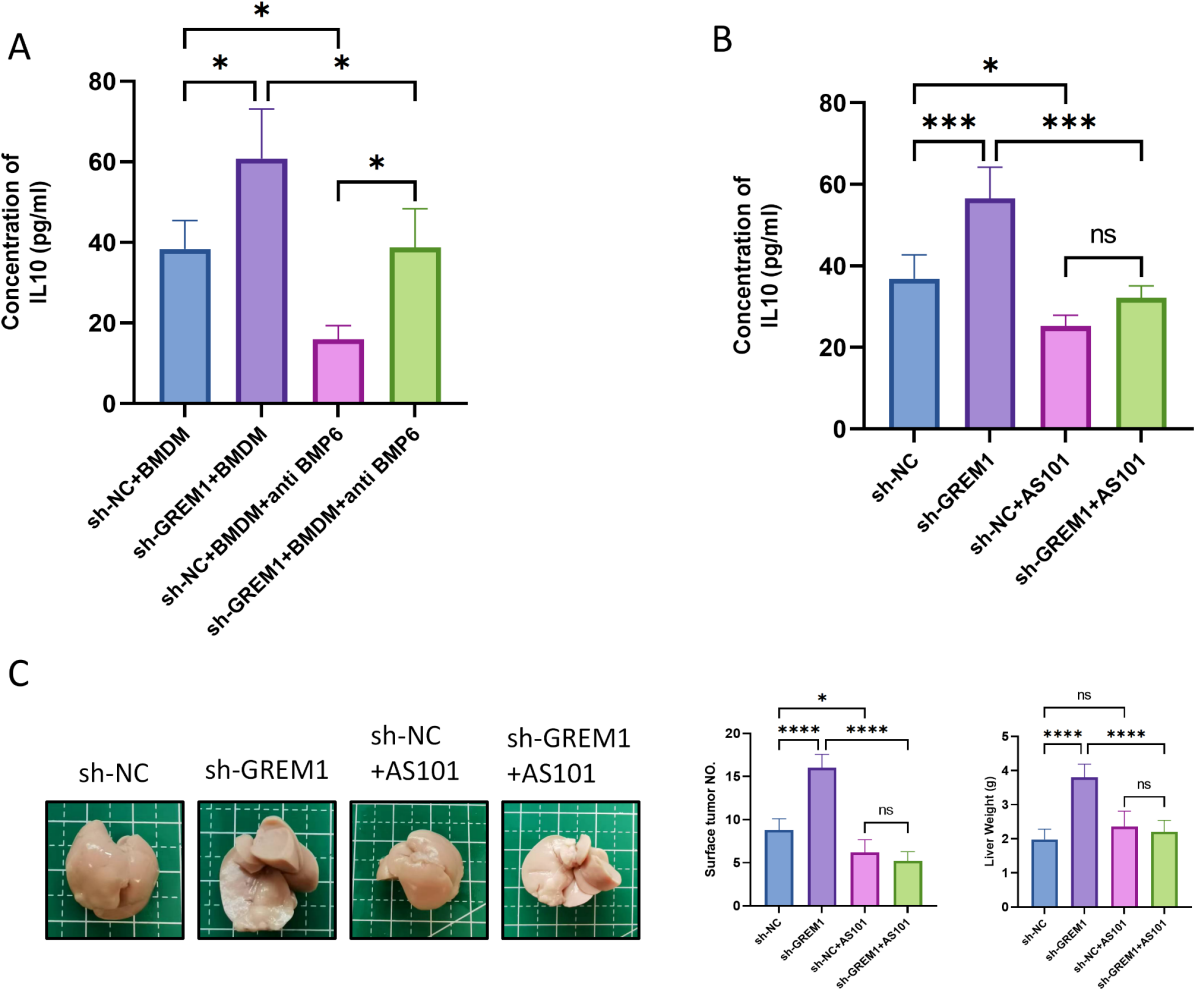
BMP6 promotes M2 polarization by inducing IL-10 release from macrophages. (**A**) The levels of IL-10 in the supernatant of BMDMs after co-cultured with MC38/sh-GREM1(sn), MC38/sh-NC (sn), MC38/sh-GREM1(sn) + anti-BMP6 neutralizing antibodies, MC38/sh-NC (sn) + anti-BMP6 neutralizing antibodies measured by ELISA. (**B**) Representative histograms of the concentration of IL-10 in liver metastases from CRLM model mice treated with AS101 or not. (**C**)The detectable tumor numbers in liver metastasis from the sh-NC or sh-GREM1-injected mice treated with AS101 or not

In order to explore how BMP6 affects macrophage polarization, We investigated the IL-10 level in BMDM supernatant of different intervention groups. The consequences exhibited that inhibiting the content of BMP6 could decrease the content of IL-10 in the supernatant of culture medium(Fig. 7A). IL-10 is one of the important cytokines secreted by macrophages, which can promote tumor progression. In vivo, we studied the effect of IL-10 inhibitor AS101 on CRLM production in mice. We used 10 µg AS101 to inhibiting IL-10 expression in mice. The inhibitory effect of AS101 on IL-10 was confirmed by ELISA detection of liver metastases(Fig. 7B). The number of liver metastases and liver weight in sh-GREM1 + AS101 group was significantly less than that in sh-GREM1 group(Fig. 7C). These results indicate that BMP6 may induces M2 polarization of macrophages by inducing IL-10 release.

## Discussion

Colorectal cancer was known to be the third most common malignant tumor in the world [25, 26]. CRLM is one of the important factors affecting the mortality of colorectal cancer [4, 5]. Therefore, understanding the molecular mechanism of CRLM progression, identifying key oncogenes that regulate cancer cell migration, invasion and liver metastasis, and screening molecular markers that predict the risk of CRLM may be considered as necessary conditions for monitoring the development of CRLM and designing new treatment strategies.

In this study, we screened out that miR-455/GREM1 axis plays a critical role during the process of liver metastasis of colorectal cancer by analyzing three datasets in GEO database, and creatively proposed that miR-455 negatively regulates GREM1 and then promote the proliferation and migration of intestinal cancer cells.

More and more evidences confirm that miRNAs have important influence on the growth and metastasis of cancer, and are related to the development and progression of cancer. Previous studies have indicated that miR-455, as a tumor-promoting factor, promotes cell proliferation and migration in various tumors. Our study found that miR-455 increased expression in hepatic metastases of colorectal cancer and promoted the growth and migration of colorectal cancer cells by targeting GREM1.

GREM1 is known to be a member of BMP antagonist family and plays an important role in many physiological and pathological processes. Previous research suggested that GREM1 bind preferentially to BMP2 and BMP4 compared with BMP7 [27], It has also been found that GREM1 can antagonize BMP6 [28]. We found that BMP6 was highly expressed in LM tissues, but there was no significant difference between BMP2 and BMP4 expression in LM, PT and PC tissues. BMP6 has been reported to promote IL-10 specific macrophage M2 polarization in renal cell carcinoma, which coincides with our findings in colorectal carcinoma [29]. In previous studies, GREM1 plays different roles in the development of different tumor cells. High expression of GREM1 promotes the growth and proliferation of tumor cells in breast cancer and glioma [20, 30], but inhibits the development of tumors in osteosarcoma and pancreatic ductal adenocarcinoma [21, 22]. Lan L et al. found that GREM1 is necessary to maintain the heterogeneity of pancreatic cancer cells, and the deletion of GREM1 greatly increases the probability of liver metastasis of pancreatic ductal adenocarcinoma [21]. The role of GREM1 in colorectal cancer is controversial. Jang BG et al. found that the expression of GREM1 in colorectal cancer is lower than that in matched normal mucosa. High expression of GERM1 is closely related to low lymphovascular infiltration and better prognosis [24]. On the contrary, Kobayashi H found that high expression of GREM1 is related to poor survival rate, and GREM1 neutralizing antibody reduces the growth of CRC tumors [31].

Our results illustrate that knocking down GREM1 can promote the proliferation and migration of colorectal cancer cells. In vivo, Deletion of GREM1 can promote the occurrence of liver metastasis of colorectal cancer. Through bioinformatics analysis, we explored that PI3K/AKT pathway is a possible downstream pathway of miR-455/GREM1 axis, which plays an important role in the process of liver metastasis of intestinal cancer. In vitro, we found that knocking down the expression of GREM1 increased the expression of p-PI3K and p-AKT, but had no obvious effect on the expression of PI3K and AKT. These results indicate that low GREM1 can promote the phosphorylation of PI3K/AKT pathway, and then promote the proliferation and migration of colorectal cancer cells. Previous studies have also found that most factors affecting CRC progression and metastasis play a role through PI3K/AKT pathway. Tumor-associated macrophages play an important role in promoting the occurrence and development of tumors. Immune infiltration analysis demonstrated that the expression of GREM1 was positively correlated with M1 polarization of macrophages and negatively correlated with m2 polarization of macrophages. Our study found that the expression of BMP6 increased after GREM1 knockout, which in turn promoted the release of IL-10 from macrophages and promoted the M2 polarization of macrophages. However, the effect of miR-455/GREM1 axis on CRLM has been preliminary explored in this study, but the specific mechanism of miR-455/GREM1 axis on PI3K/AKT pathway is still unclear, and the exact mechanism of macrophage polarization affected by knockout of GREM1 needs further discussion.

## Conclusions

In conclusion, miR-455/GREM1 axis plays an irreplaceable role in the development of CRLM. Knocking down GREM1 activates PI3K/AKT phosphorylation and promotes macrophage M2 polarization by increasing BMP6 expression, thus promoting the proliferation and migration of colorectal cancer in vitro and promoting the occurrence of liver metastasis of colorectal cancer in vivo. miR-455 and GREM1 are important factors in the process of liver metastasis of colorectal cancer.

### Electronic supplementary material

Below is the link to the electronic supplementary material.

Supplementary Material 1

Supplementary Material 2

## Data Availability

The data that support the findings of this study are available from the corresponding author upon reasonable request.
