# Supplementary figures and images for: miR-455/GREM1 axis promotes colorectal cancer progression and liver metastasis by affecting PI3K/AKT pathway and inducing M2 macrophage polarization
Source: Cancer Cell Int. 2024 Jul 5;24:235. doi: 10.1186/s12935-024-03422-1 (PMC11225248; doi:10.1186/s12935-024-03422-1)

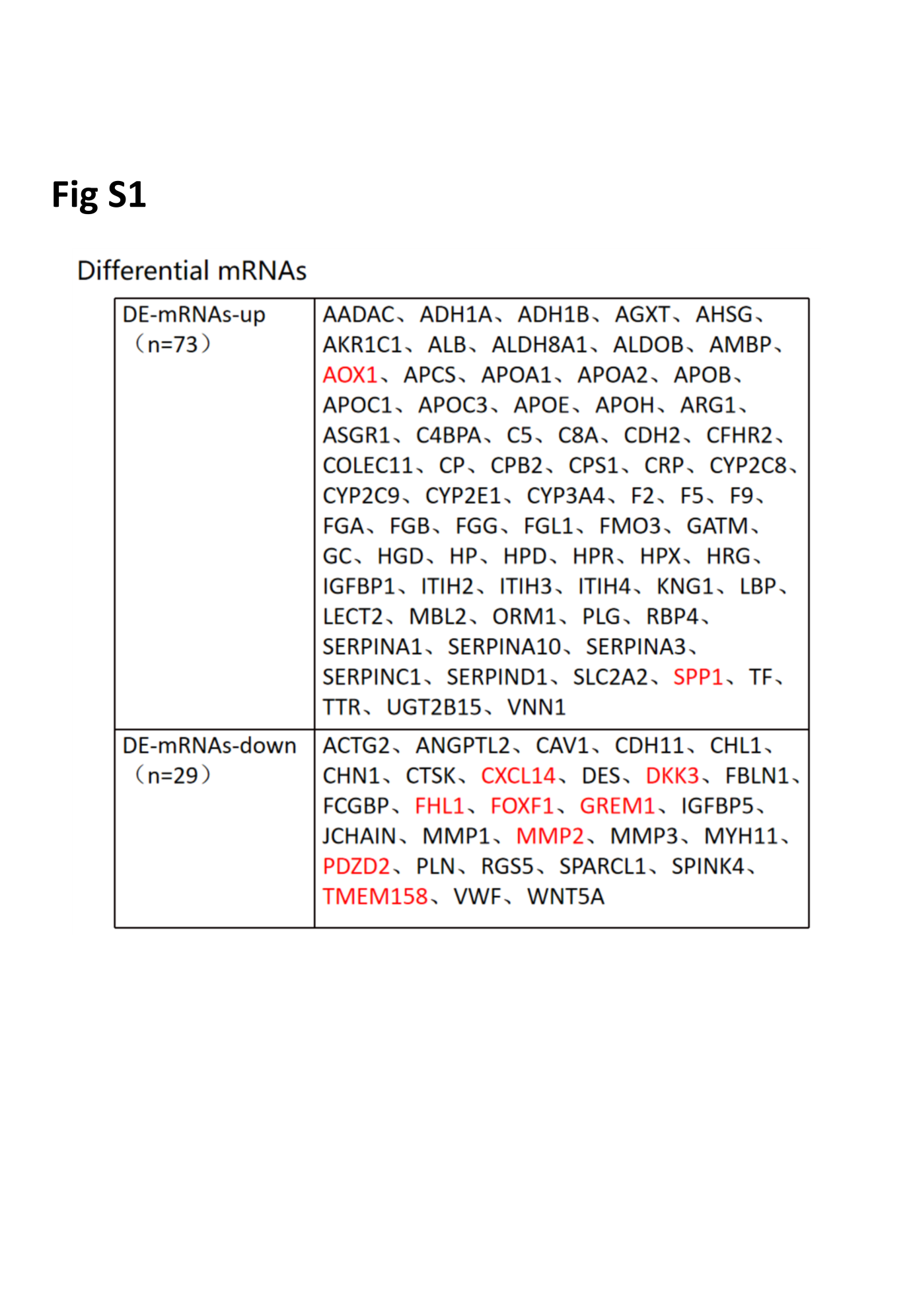


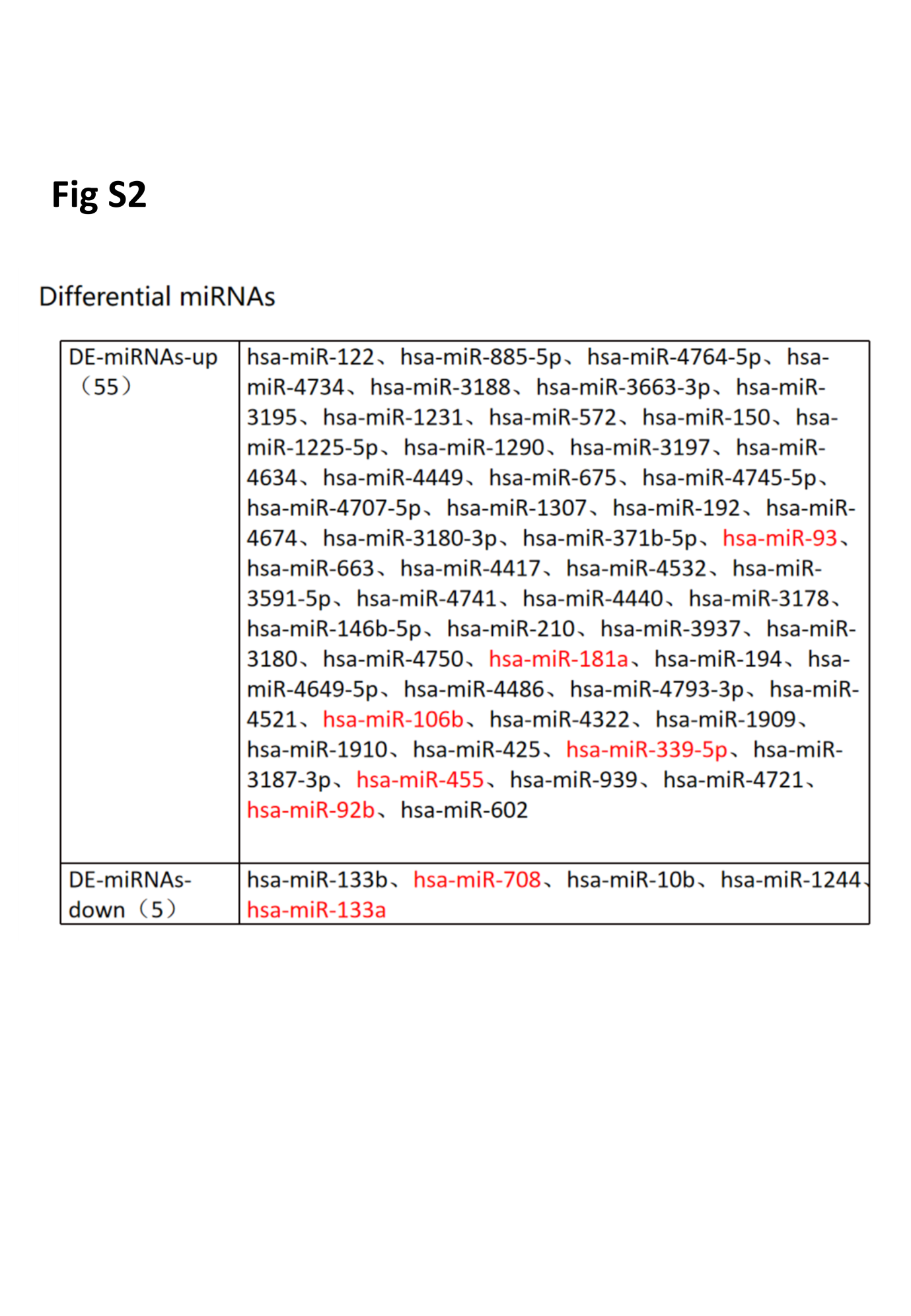

Supplement: Supplementary file 1 — Supplementary Material 1 [file 12935_2024_3422_MOESM1_ESM.docx]
