# Supplementary material for: miR-455/GREM1 axis promotes colorectal cancer progression and liver metastasis by affecting PI3K/AKT pathway and inducing M2 macrophage polarization
Source: Cancer Cell Int. 2024 Jul 5;24:235. doi: 10.1186/s12935-024-03422-1 (PMC11225248; doi:10.1186/s12935-024-03422-1)
Supplement: Supplementary file 2 — Supplementary Material 2 [file 12935_2024_3422_MOESM2_ESM.docx]

**Table S1. The gene-specific primer sequences.**

| GREM1 | Forward Primer: 5’-GGAGCCCTGCATGTGACG-3’ |
| --- | --- |
|  | Reverse Primer: 5’-GAAGCGGTTGATGATGGTGC-3’ |
| miR-455 | Forward Primer: 5′-ACACTCCAGCTGGGTATGTGCCTTTGGACT-3′ |
|  | Reverse Primer: 5′-CTCAACTGGTGTCGTGGAGTCGGCAATTCAGTTGAGCGATGTAG-3′ |
| CD86 | Forward Primer: 5’-ATGGGCTCGTATGATTGT-3ʹ |
|  | Reverse Primer: 5’-CTTCTTAGGTTTCGGGTG-3ʹ |
| NOS2 | Forward Primer:5’-CACAGCAATATAGGCTCATCCA-3’ |
|  | Reverse Primer: 5’-GGATTTCAGCCTCATGGTAAAC-3’ |
| ARG1 | Forward Primer:5’-AGGCGCTGTCATCGATTTCT-3’ |
|  | Reverse Primer: 5’-TGGAGTCCAGCAGACTCAAT-3’ |
| MRC1 | Forward Primer:5’-GTTCACCTGGAGTGATGGTTCTC-3 |
|  | Reverse Primer: 5’-ACCACCCTGTTGCTGTAGCCAA-3’ |
| BMP2 | Forward Primer: 5′-CACACAGGGACACACCAACC-3′ |
|  | Reverse Primer: 5′-CAAAGACCTGCTAATCCTCAC-3′ |
| BMP4 | Forward Primer: 5′-GAGGAGTTTCCATCACGAAGA-3′ |
|  | Reverse Primer: 5′-GCTCTGCCGAGGAGATCA-3′ |
| BMP6 | Forward Primer: 5′-GCTCAACCGCAAGAGCCTTC-3′ |
|  | Reverse Primer: 5′-TGTCGTACTCCACCAGGTTC-3′ |
| GAPDH | Forward Primer: 5’- GTCTCCTCTGACTTCAACAGCG-3’ |
|  | Reverse Primer: 5’-ACCACCCTGTTGCTGTAGCCAA-3’ |
